# Population genetic structure and intraspecific genetic distance of *Periplaneta americana* (Blattodea: Blattidae) based on mitochondrial and nuclear DNA markers

**DOI:** 10.1002/ece3.5777

**Published:** 2019-11-04

**Authors:** Jinnan Ma, Jinhua Liu, Yongmei Shen, Zhenxin Fan, Bisong Yue, Xiuyue Zhang

**Affiliations:** ^1^ Key Laboratory of Bio‐resources and Eco‐environment Ministry of Education College of Life Sciences Sichuan University Chengdu China; ^2^ Sichuan Key Laboratory of Conservation Biology on Endangered Wildlife College of Life Sciences Sichuan University Chengdu China; ^3^ Sichuan Key Laboratory of Medicinal American Cockroach Sichuan Gooddoctor Pharmaceutical Group Chengdu China

**Keywords:** genetic distance, genetic diversity, geographic genetic structure, *Periplaneta americana*

## Abstract

The American cockroach (*Periplaneta americana*) is a globally invasive pest that can cause significant economic loss and threaten human health. Although it is abundant and lives in close proximity to humans, few studies have investigated the genetic diversity of *P. americana*. Our study analyzed 1,053 *P. americana* and other *Periplaneta* species' samples from different locations in China and the United States. A traditional tree‐based method using 17 unique mitochondrial COI haplotypes of *P. americana* and 20 haplotypes of the other *Periplaneta* species accurately identified *P. americana* with a barcoding threshold of 5.1%. To identify the population genetic structure of *P. americana*, we investigated *wingless* gene and pooled them with obtained mtDNA data for a combined analysis. Although the genetic diversity of the USA group was relatively higher than the China group, the number of haplotypes and alleles of both groups was small. The analysis of molecular variance (AMOVA), intraspecific phylogeny, and haplotype networks indicated that *P. americana* had very little global genetic differentiation. The weak geographic genetic structure might reflect the human‐mediated dispersal of *P. americana*. Despite no apparent phylogeographic assignment of mtDNA and nuclear lineages was observed in both BI trees, the integrated COI sequence data identified four distinct *P. americana* haplotype groups, showing four ancient maternal lineages of *P. americana* in China and the United States.

## Graphical abstract

Here, we assessed genetic diversity and genetic structure of *Periplaneta americana* specimen from 18 sites in China using mtDNA and nDNA markers and then compared the genetic pattern of *P. americana* in China with the Americas. Although no clear pattern of genetic structure was detected within *P. americana*, four mitochondrial lineage units of *P. americana* showed clear genetic signatures of ancestor haplotypes. Additionally, we analyzed the COI barcode region, using traditional tree‐based, ABGD, and GMYC species delimitation methods to determine the intraspecific genetic distance threshold of *P. americana* in a larger range of sampling. Compared to GMYC and ABGD approaches, the traditional tree‐based method could accurately and effectively identify *P. americana* from other five *Periplaneta* cockroaches species with barcoding threshold 5.1%.

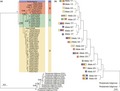

## 1 INTRODUCTION

Cockroaches are members of the order Blattodea, containing at least 4,600 species and 460 genera (Beccaloni, 2014). *Periplaneta* belongs to the subfamily Blattinae within the family, Blattidae. There are approximately 53 species of the *Periplaneta* genus (Beccaloni, 2014), and most *Periplaneta* species are not closely associated with humans. Only a relatively small number of *Periplaneta* cockroaches are known as pests and are dominant species in urban environments, including *P. americana*, *P. fuliginosa*, *P. australasiae*, *P. japonica*, and *P. brunnea* (Roth & Willis, 1960). They are considered to be a mechanical vector of various pathogenic organisms and can cause health problems such as asthma and allergies (Bell, Roth, & Nalepa, 2007). Considering their high reproductive ability and habitat adaptability, *P. americana* is the most abundant and widely distributed species within *Periplaneta* (Roth & Willis, 1960).

Accurate taxonomic identification is the cornerstone of developing management strategies for invasive species. However, traditional methods to identify *P. americana* and other *Periplaneta* based on morphological characteristics have been problematic due to highly similar external morphology (Evangelista, Buss, & Ware, 2013), high degree of polymorphism between adults and juveniles (Evangelista, Bourne, & Ware, 2014), and sexual dimorphism (Che, Gui, Lo, Ritchie, & Wang, 2017; Evangelista et al., 2014). Therefore, it would be indispensable to apply a rapid and effective molecular identification method, such as using the mitogenome, to complement the morphological taxonomy of *P. americana*. The mitogenome is characterized by its maternal inheritance, nonintrons, and rapid evolution (Cameron, 2014). The application of a short standardized mitochondrial cytochrome c oxidase I (COI) 5′ region has been highly successful in a wide range of insect taxa (Che et al., 2017; Talavera, Muñoz‐Muñoz, Verdún, & Pagès, 2017; Versteirt et al., 2015). Several different methods using molecular makers have been put forward to identify distinct species. Traditional DNA barcoding calculates intra‐/interspecific genetic distances and constructs neighbor‐joining (NJ) tree for species delimitation. The clustered clades in a phylogenetic tree and the existence of the barcoding gap are interpreted as distinct species (Ni, Li, Kong, Huang, & Li, 2012). In addition to traditional barcoding analysis (NJ analysis), generalized mixed Yule‐Coalescent (GMYC) is also a popular approach for species identification based on single‐locus data, which estimates species boundaries from branching rates in a phylogenic tree (Fujisawa & Barraclough, 2013). Another method, automatic barcode gap discovery (ABGD), automatically sorts sequences into hypothetical species based on the barcode gap (Puillandre, Lambert, Brouillet, & Achaz, 2012). A combination of methods can be helpful to evaluate the efficiency of DNA barcoding for *P. americana* identification.

Intraspecific diversity studies, using both mitochondrial and nuclear markers, enable us to evaluate the population genetic structure and genetic diversity of a species (Ferronato et al., 2019; Johnson, Morton, Schemerhorn, & Shukle, 2011; Roman, 2006; Wang et al., 2009). Although *P. americana* is an urban pest worldwide, there are limited studies on the genetic variety and population structure of *P. americana*. One study, based on multiple samples from eastern United States, suggested that “*P. americana* individuals from three or more historically isolated geographic populations are now effectively merged into a single global gene pool” (von Beeren, Stoeckle, Xia, Burke, & Kronauer, 2015). Understanding the population structure and genetic variety of invasive species across different continents may help to comprehend the possible pathways and invasion history of *P. americana*, but to our knowledge, there has been no examination of the genetic variation of *P. americana* groups in China.

There are two objectives that are pivotal to this study. Firstly, the COI barcode region of a broader geographic range of *P. americana* and other *Periplaneta* species collected in China and the United States was analyzed using traditional tree‐based, ABGD, and GMYC species delimitation methods. Our intention was to observe which of these methods best corresponds to morphological species concepts in *Periplaneta* and the levels of intraspecific rate of genetic variation that exists within *P. americana*. Our second objective was to assess the genetic diversity and genetic structure of *P. americana* specimens from 18 sites in China using both *wingless* and COI markers. We then integrated the previously registered *P. americana* sequences (von Beeren et al., 2015) into our genetic data and reanalyzed the dataset in order to compare the phylogenetic structure of *P. americana* in China and the Americas.

## 2 MATERIALS AND METHODS

### 2.1 Samples collection

**Figure 1:**
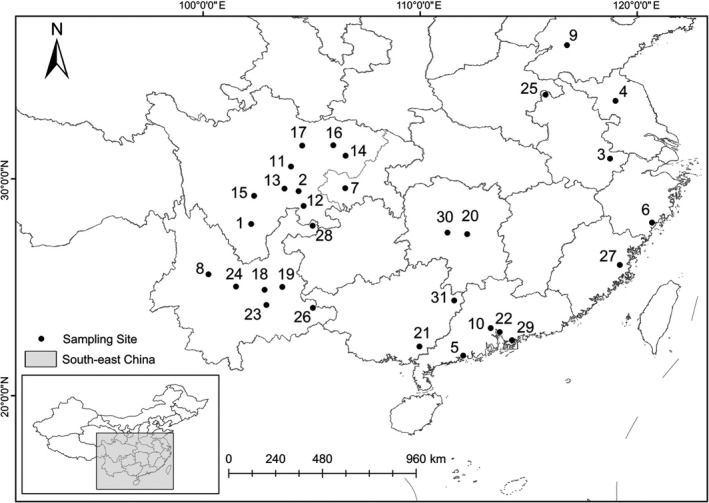
Distribution and sampling localities of cockroaches analyzed in this work. Numbers for sampling localities are as indicated in Table S1

We collected 853 *Periplaneta* specimens (including *P. americana*, *P. fuliginosa*, *P. australasiae*, and *P. brunnea*) from 31 sampling locations in China (Table S1, Figure 1). Species used in the phylogenetic analyses, sampling ID, and GenBank accession numbers are available on Dryad: https://doi.org/10.5061/dryad.280gb5mm1. Morphological species identification was carried out by using the taxonomic keys for the cockroaches (Liu, Zhu, Da, & Wang, 2017; Robinson, 2005). Specimens of nymphs were excluded for lack of discernible morphological characters.

### 2.2 DNA extraction, amplification, and sequencing

Total DNA was extracted from muscle tissue using the TsingKe Genomic DNA kit (TsingKe). The partial sequences of mitochondrial COI gene (658 bp) and nuclear *wingless* gene (378 bp) were amplified by PCR with the primers LCO1490/HCO2198 (Folmer, Black, Hoeh, Lutz, & Vrijenhoek, 1994) and wg550F/wgcockR (von Beeren et al., 2015). Not all the COI genes of specimens were successfully sequenced by Folmer's universal primers because PCR always co‐amplified a large number of pseudogenes, which can be due to bimodal sequencing (Song, Moulton, & Whiting, 2014). We designed a set of primers to eliminate nontarget DNA sequencing. The primers QF/QR, HF/HR, and AUSF/AUSR were designed specifically for *P. americana*, *P. fuliginosa*, and *P. australasiae*, respectively (Table 1). Our specific primers were effective in preventing failed amplifications. PCRs were set up in 25 μl reaction volumes with 100 ng of total DNA, 0.3 μl of 5 U/μl Taq DNA polymerase (Takara), 1 μl of 2.5 mM dNTPs, 2.5 μl of 10 × PCR buffer (+Mg^2+^; Takara), and 0.5 μl of 25 μM respective primers. Successfully amplified fragments were purified using the DNA agarose gel extraction kit (TsingKe) and sequenced by ABI PRISM 3730 DNA sequencer (TsingKe Biotechnology Company, Chengdu, China).

**Table 1:** Primers used in this study

Gene	Primer	Primer sequence	Anneal temperature (°C)	Extension time (s)
*wingless*	wg550F	5′‐ATGCGTCAGGARTGYAARTGYCAYGGYATGTC‐3′	64	15
	wgcockR	5′‐AACATGCACGCACACCTCTGCACCACGGACACC‐3′		
COI	LCO1490	5′‐GGTCAACAAATCATAAAGATATTGG‐3′	55	30
	HCO2198	5′‐TAAACTTCAGGGTGACCAAAAAATCA‐3′		
COI	Q[F]	5′‐CTCAGCCATTCTACTAACTTTGC‐3′	55	90
	Q[R]	5′‐CTATAATAGGAGATGCTCTGTCTTG‐3′		
COI	H[F]	5′‐TTACCTTCGAATCTGTTATGC‐3′	55	72
	H[R]	5′‐GCTGATGTAAAATAAGCTCGTG‐3′		
COI	AUS[F]	5′‐ATCAATTTCCATATTTGGCTT‐3′	55	60
	AUS[R]	5′‐GCTGATGTAAAATAAGCTCGTG‐3′		

### 2.3 Marker summary statistics and intrapopulation genetic diversity

In total, mitochondrial COI sequences of 853 cockroach specimens (including 563 *P. americana* specimens, 235 *P. fuliginosa* specimens, 52 *P. australasiae* specimens, and 3 *P. brunnea* specimens) were successfully amplified. Forty‐eight individuals from each of *P. americana* COI haplogroups (results from phylogenetic analyses based on *P. americana* COI haplotypes) were selected for *wingless* amplification to corroborate the patterns of genetic structure, differentiation, and divergence of *P. americana* populations. The dataset, which included 853 COI and 48 *wingless* sequences, was submitted to GenBank (https://www.ncbi.nlm.nih.gov/) under the accession numbers MF149138–MF149711, MH184206–MH184379, and MK658782–MK658829. In addition to the new data we collected, 200 COI and 68 *wingless* sequences from GenBank for *Periplaneta* species were added for analysis (a detailed list of previous GenBank records of *Periplaneta* spp. is available on Dryad repository: https://doi.org/10.5061/dryad.280gb5mm1). We grouped the *P. americana* samples by country. The groups from China and the United States included 564 and 195 specimens, respectively. DNA SeqMan (DNAStar Inc.) was used to assemble sequences. Sequences were aligned using the program CLUSTAL W in MEGA 5.0 (Tamura et al., 2011). DNA sequences were checked visually and translated to DNA codons to avoid pseudogenes (Zhang & Hewitt, 1996). The number of conserved, variable, parsimony‐informative sites and singletons were assessed in MEGA 5.0. DnaSP 5.10.01 (Librado & Rozas, 2009) was used to calculate haplotype distribution, haplotype diversity (*H*_d_), and nucleotide diversity (*P_i_*).

For the nuclear marker, direct sequencing of PCR products indicated that many individuals were heterozygous. Alleles of heterozygous individuals were identified with DnaSP 5.10.01 (Librado & Rozas, 2009) by applying PHASE algorithms. Only three individuals (KM591680.1, KM591631.1, and KM591621.1) were omitted from population genetic structure analyses due to their inferred alleles with low probability (*p* < .8). All genotype information for each sample was presented on Dryad repository: https://doi.org/10.5061/dryad.280gb5mm1.

### 2.4 Neighbor‐joining clustering and species delimitation approaches

The neighbor‐joining (NJ) tree was performed with bootstrap analysis (1,000 replicates) in MEGA 5.0 based on the Kimura 2‐parameter (K2P) distance model. Furthermore, intra‐/interspecific genetic distances with the same model were calculated.

In addition to traditional barcoding analysis (NJ analysis), we used GMYC and ABGD approaches to examine the congruence of OTUs (operational taxonomic units). GMYC is a tree‐based approach for the delimitation of species. An ultrametric tree was produced using BEAST v1.10.4 (Suchard et al., 2018) under the following parameters: GTR + G substitution model with four gamma categories; lognormal relaxed molecular clock model; Yule process prior; and 400,000,000 generations sampling every 20,000 generations. The remaining settings were left as defaults. The distribution of log‐likelihood scores and trace files of runs were evaluated using Tracer v1.6. A maximum clade credibility tree was constructed in TreeAnnotator v1.10.4. The outcome tree was read into the “splits” R package and run with the single‐threshold GMYC method in R v3.5.2 project.

The ABGD (Puillandre et al., 2012) is a model‐based method for inferring putative species. The pairwise genetic distances were ranked from smallest to largest to detect the barcoding gap. ABGD uses the first significant gap beyond one‐side confidence limit to partition the data and then recursively applies inference of the limit and gap detection to obtain finer partitions until no further partition can be detected. This method was implemented online (http://wwwabi.snv.jussieu.fr/public/abgd/) with default parameters (*P*_min_ = 0.001, *P*_max_ = 0.1, Steps = 10, number of bins = 20, distance method = Kimura).

### 2.5 Population genetic structure

Calculation of Fixation Index (*F*_ST_) and analysis of molecular variance (AMOVA) were performed using ARLEQUIN v3.5 (Excoffier & Lischer, 2010). The *N*_m_ values were calculated to measure population contact as migrating reproductive individuals per generation and the equation used was *N*_m_ = (1 − *F*_ST_)/4*F*_ST_. These analyses were conducted to assess genetic variation according to geographic distribution. The spanning network of COI haplotypes was constructed using TCS 1.21 at 95% confidence level (Clement, Posada, & Crandall, 2000) to study the relationships between haplotypes and their geographic distribution.

Phylogenetic relationships between *P. americana* groups based on nuclear and mitochondrial DNA were estimated using a Bayesian approach. The best‐fitting model for BI analysis was calculated using Modeltest ver. 3.7 (Posada & Crandall, 1998) under Akaike information criterion (AIC; Akaike, 1974). The best‐fit substitution model selected was GRT + I+G (*N*_ST_ = 6, Rates = gamma) for COI sequences and HKY (*N*_ST_ = 2, Rates = equal) for *wingless* sequences. Subsequently, BI analysis of nucleotides was implemented with MrBayes 3.2.2 (Ronquist & Huelsenbeck, 2003), where we ran four chains in parallel for 10,000,000 generations. The phylogenetic trees were visualized in FigTree v1.4.0 (Rambaut, 2007).

## 3 RESULTS

### 3.1 Variations in nucleotide sequences

In total, we analyzed the genetic variability of *P. americana* mitochondrial COI sequence for 18 sampling sites in China (17 were collected from China for this study and the PACN from GenBank), and the results were compared with the *P. americana* group from the United States (Table S1). The nucleotide sequence (658‐bp segment) of the COI gene in this study had no stop codons, no unusual amino acid substitutions or internal sequence deletions, indicating that all sequences were functional mitochondrial sequences and not nuclear pseudogenes. The COI sequences of 759 specimens of *P. americana* yielded 17 haplotypes, of which haplotypes one to nine were newly defined in this study. The number of *P. americana* haplotypes per sampling site ranged from one to eight (Table S2). Of these, four haplotypes were shared by at least two sampling sites, with the most frequent haplotype, PAH1, present in sites collected from China as well as in the group from the United States. There were nine haplotypes characteristic for the Chinese group (PAH2 to PAH10), whereas haplotypes PAH11 to PAH17 were only found in the group from the United States. All 17 haplotypes showed 608 conserved sites, 50 variable sites, 22 parsimony‐informative sites, and 28 singleton sites. The average nucleotide composition of those sequences was 33.1% T, 18.8% C, 32.0% A, and 16.0% G. A + T (65.1%) was present in a much higher proportion than G + C (34.8%), as it is usual for insects (Simon, Buckley, Frati, Stewart, & Beckenbach, 2006). Molecular diversity indices of *P. americana* are given in Table S2. The haplotype diversity (*H*_d_) and nucleotide diversity (*P_i_*) within each sampling site in China ranged from 0.0 to 0.625 and from 0.0 to 0.01179, respectively. Among them, almost half of all Chinese sampling sites showed only one haplotype, and the haplotype diversity and nucleotide diversity of these sites were zero. When the *P. americana* samples from China were considered as a single group, they indicated relatively low haplotype diversity (0.375) and nucleotide diversity (0.00659) when compared to samples from the United States (Table S2).

The fragments of the nuclear gene *wingless* of several *P. americana* individuals were also sequenced in this study (*N* = 48). Inclusion of 68 additional *wingless* sequences from GenBank from the United States produced a final alignment of 378 bp for 116 individuals. A total of seven distinct *wingless* alleles were identified with high probability (Table 2). Of the 378 nucleotide positions, ten parsim‐info positions were observed (2.6%). For the nuclear marker, a comparison of *H*_d_ and *P_i_* values between the Chinese and United States *P. americana* groups is shown in Table S2. The group from the United States had a slightly higher level of haplotype diversity and nucleotide diversity than the Chinese group, which is consistent with mitochondrial gene analysis. Specimens from China and the United States exhibited comparable levels of Hd and Pi values in both markers.

**Table 2:** Variable positions of seven alleles of *wingless* gene sequence for *Periplaneta americana*

Alleles	Nucleotide position beginning from 5′ end											Allele frequencies
	55	61	85	124	138	151	184	298	331	355	376	
Allele 1	A	C	C	C	G	A	A	C	G	T	T	0.146018
Allele 2	A	C	C	C	G	C	A	C	G	T	T	0.261062
Allele 3	A	C	C	C	G	C	A	C	A	T	T	0.070796
Allele 4	A	C	C	T	A	C	G	C	G	C	C	0.230088
Allele 5	A	C	G	C	G	C	G	T	G	C	C	0.106195
Allele 6	A	T	C	C	G	C	A	C	G	T	T	0.004425
Allele 7	C	C	G	C	G	C	A	C	G	T	T	0.181416

### 3.2 COI marker barcoding

**Figure 2:**
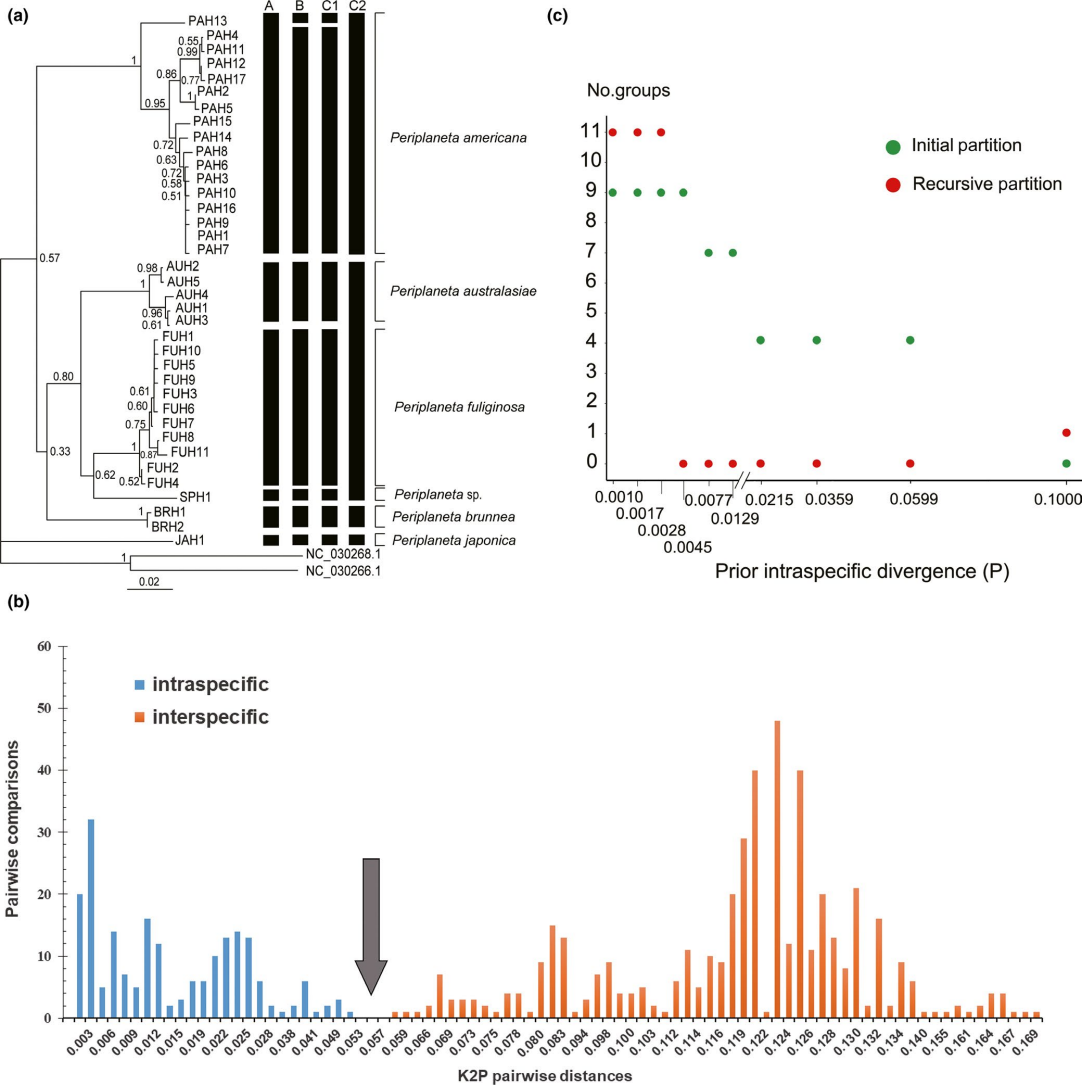
(a) The NJ tree constructed by COI haplotype sequences of *Periplaneta* based on Kimura 2‐parameter distances. The results of different species delimitation methods: traditional tree‐based (column A), GMYC model (column B), ABGD (primary partitions with *p* value between 0.77% and 1.29%; column C1), and ABGD (primary partitions with *p* value between 2.15% and 5.99%; column C2) are indicated at the right edge of the tree. The horizontal bars illustrate the species delimitations for each individual method. The numbers in the nodes represent the bootstrap values with 1,000 replications. Taxon names and locality of individuals with different haplotypes are as indicated in Table S1. (b) Frequency distribution histograms of all intraspecific and interspecific K2P pairwise distances for the COI gene, exhibiting barcoding gap (gray arrow). (c) Automatic partition of *Periplaneta* species based on COI sequence. The number of groups inside the partition (initial and recursive) of each given prior intraspecific divergence value is reported

The data matrices of *Periplaneta* spp., which contained 17 unique COI haplotypes of *P. americana* and 20 haplotypes of the other *Periplaneta* species (Table S1), were included for species delimitation analysis. Traditional DNA barcoding, ABGD, and GMYC methods were applied to examine the consensus of OTUs. Since traditional species delimitation is mainly determined based on genetic gaps, neighbor‐joining (NJ) analysis was first used for this purpose. *P. americana* and a number of other *Periplaneta* species (*P. brunnea*, *P. fuliginosa*, *P. australasiae*, *P. japonica*, and *P. sp*) clustered together with a high support value (Figure 2a). Different morphospecies can be isolated in the separate clusters. *P. americana* was a sister group of *P. brunnea*, *P. fuliginosa*, *P. australasiae*, and *P. sp*, whereas *P. japonica* was the sister group to the remaining *Periplaneta* species. Genetic distances were then calculated to evaluate the levels of interspecific and intraspecific divergence for those defined species clades (Table 3; more detailed information is available on Dryad: https://doi.org/10.5061/dryad.280gb5mm1). The maximum intraspecific divergence value (5.1%) within *Periplaneta* species was observed in *P. americana*, followed by *P. australasiae* (1.9%) and *P. fuliginosa* (1.2%). Higher levels of genetic distance were found between those six *Periplaneta* species. The minimum interspecific divergence was 5.8% between *Periplaneta* sp. and *P. fuliginosa*. When comparing *P. americana* to other *Periplaneta* species, the minimum and maximum interspecific divergence values were 11.0% and 16.9%, respectively. COI analysis showed no overlap between maximum intra‐ and minimum interspecific divergence values and a barcoding gap was apparently present (Figure 2b).

**Table 3:** General barcode information and genetic variation (%) of COI barcodes haplotypes within (intra) and between (inter) *Periplaneta* species included in this study

Species	NC	COI‐intra		COI‐inter	
		Min (%)	Max (%)	Min (%)	Max (%)
*Periplaneta americana*	17	0.2	5.1	11.0	16.9
*Periplaneta fuliginosa*	10	0.2	1.2	5.8	13.2
*Periplaneta australasiae*	5	0.2	1.9	6.8	13.6
*Periplaneta brunnea*	2	0.2	0.2	9.4	12.6
*Periplaneta japonica*	1	—	—	11.2	16.9
*Periplaneta* sp.	1	—	—	5.8	13.6

Abbreviations: Min/Max, the minimum/maximum genetic distance value; NC, the number of COI sequences used in this analysis.

However, both ABGD and GMYC methods generated incongruent genetic lineages when compared with the traditional barcoding analysis (Figure 2a). The 20 recursive steps in the ABGD analysis resulted in ten different sequence partitions. The recursive partition produced one and 11 groups (=species), while four, nine, and seven groups in the initial partition (Figure 2c). Too high or too low prior intraspecific divergence would underestimate or overestimate the number of species (Puillandre et al., 2012). Therefore, we decided to report only primary partitions in the output of ABGD with *p* value between 0.77% and 5.99% (no group was predicted by recursive partitions with *p* value between 0.77% and 5.99%). *P. brunnea*, *P. fuliginosa*, *P. australasiae*, *P. japonica*, and *P. sp* can be distinguished with prior intraspecific divergence between 0.77% and 1.29%. However, *P. americana* had two genetic groups with prior genetic distance thresholds between 0.77% and 1.29%. Using the values between 2.15% and 5.99%, the species were partitioned into four groups (Figure 2a). *P. fuliginosa*, *P. australasiae*, and *P. sp*. were classified within the same OTU. GMYC model analysis yielded the same number of seven species as with the ABGD model (primary partitions with *p* value between 0.77% and 1.29%; Figure 2a; Figure S1). *P. americana* was split into two species, while the other five *Periplaneta* species were represented as a single species.

### 3.3 Phylogenetic and network analyses

**Figure 3:**
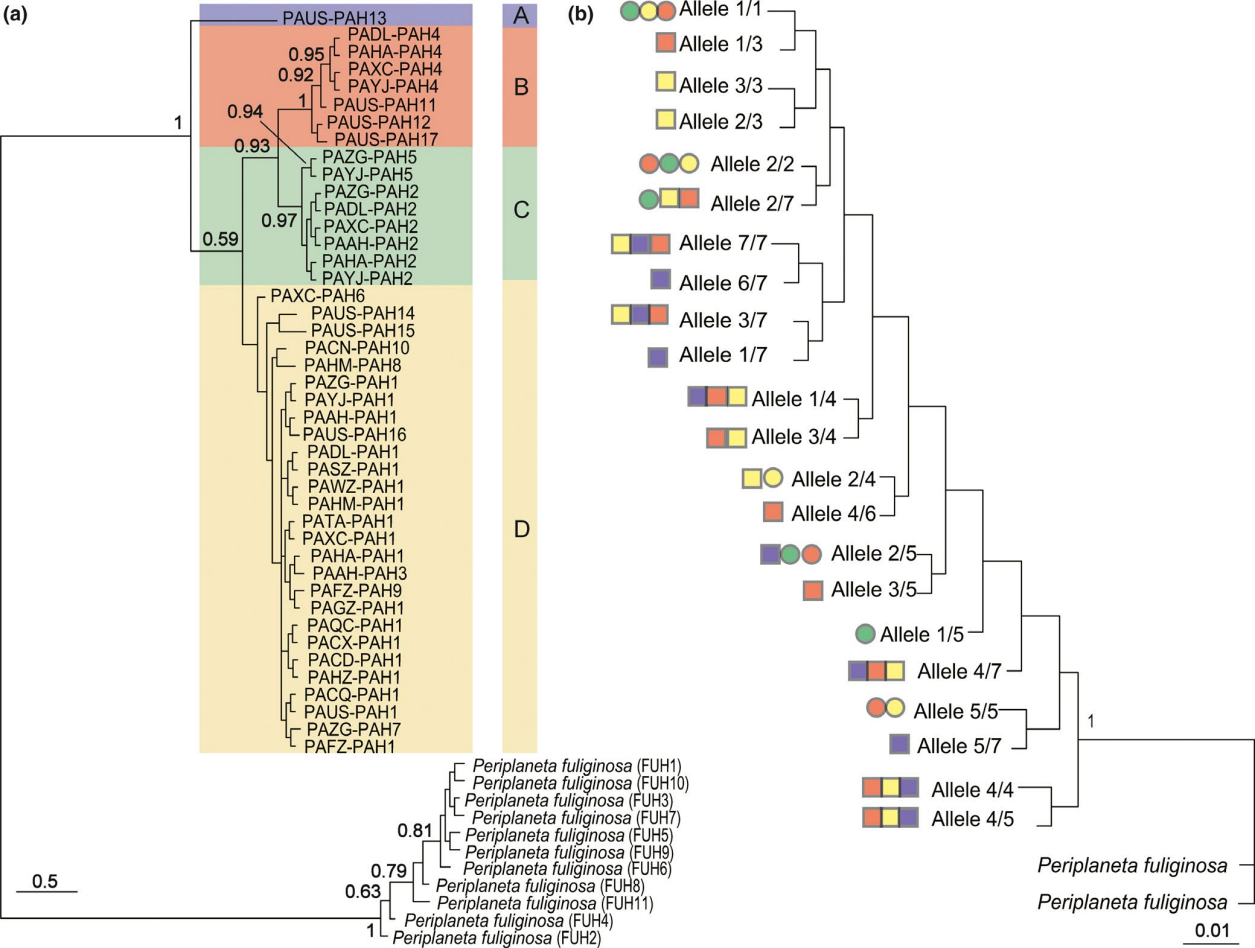
The Bayesian tree of *Periplaneta americana* from China (18 sampling sites) and the USA groups samples based on analyses of the mt COI (a) and *wingless* genes (b). The posterior probabilities exceeding 50% are shown above the nodes. The labels in COI tree include group ID and haplotype codes. Colors depict different COI haplogroups. For each individual, its two nuclear alleles are coded using different numbers. Colors on the *wingless* tree clades give the individuals belong to the same mitochondrial haplogroups. The square and the circle represent samples from the United States and China, respectively

**Figure 4:**
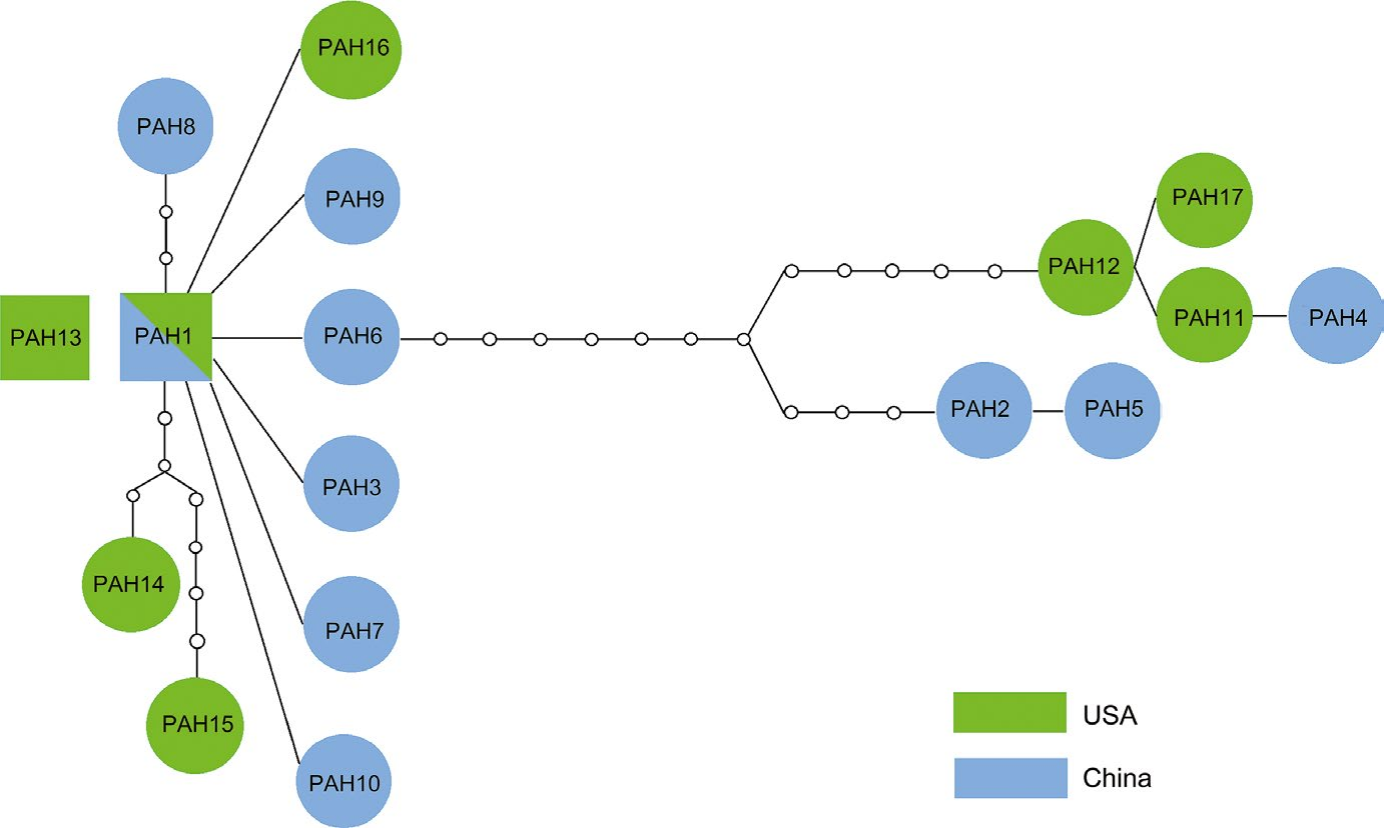
Network relationships of COI haplotypes from *Periplaneta americana*. Inferred unobserved haplotypes are shown as “o.” Each mutation step is shown as a short line connecting neighboring haplotypes

We performed phylogenetic analyses of *P. americana* using the BI method based on COI and *wingless* genes to explore the geographic relationship among the analyzed populations. *P. americana* COI haplotypes of China (18 sampling sites) and the USA groups formed four major mitochondrial clades (A, B, C, D; Figure 3a). Clades A and C only comprised of the samples from the United States and China, respectively. Clades B and D consisted of cockroach samples from both China and the United States. There was no evidence for strong geographic clustering in the phylogenetic tree. The topology of haplotype network was congruent with phylogenetic inferences and showed four haplotype clusters (Figure 4). TCS networks for each haplotype joined all but one PAH13 at the 95% confidence level. In particular, PAH1, which was shared between the United States and China, was connected to several low‐frequency haplotypes, implying that it may represent a putative ancestral haplotype. Consistent with the phylogenetic analysis, haplotypes from China (PAH2, PAH5) were closely related to haplotypes 4, 11, 12, and 17. Haplotypes PAH11, PAH12, and PAH17, which came from the United State, were generated through mutations of unique haplotype from China (PAH6).

Phylogenetic relationships obtained for the *wingless* gene were compared with phylogenetic relationships obtained with mtDNA COI data (Figure 3b). Individuals collected from China grouped not only with the other samples from China but also with samples from the United States in many separate, weakly supported small clades. Specimens from four major COI haplogroups were mixed, showing that *P. americana* was a global panmictic group with high rates of gene flow among different geographic regions.

### 3.4 Population genetic structure

Using the dataset of mtDNA, genetic divergence (*F*_ST_) and per‐generation migration rate (*N*_m_) between pairs of the 18 Chinese sampling sites were computed (data are available on Dryad: https://doi.org/10.5061/dryad.280gb5mm1). *F*_ST_ ranged from −0.250 to 1.000. A comparison of 64 of the 153 pairs of sampling sites showed no significant genetic differentiation (*p* > .05), implying that more than 50% of the pairs of sampling sites formed one genetic group. These results suggest that the 18 sampled areas of *P. americana* lacked genetic structure, which agreed with the phylogenetic and network analyses. When all Chinese specimens were grouped (18 sampling sites) to compare pairwise *F*_ST_ values with the USA samples, significant differences in haplotype and allele frequencies at both the COI (*F*_ST_ = 0.335, *p* < .05) and *wingless* (*F*_ST_ = 0.266, *p* < .05) loci were observed. Mean *F*_ST_ values within the 18 Chinese sampling sites (0.16 ± 0.18) based on the COI gene were significantly lower than that between China and the USA groups. Despite that, the American and Chinese groups both shared the same clusters and haplotype (Figure 3a).

Furthermore, an AMOVA on the COI marker including the pooled 18 China site samples and the one USA group sample indicated that a majority of nucleotide diversity (60.08%, *p* < .05) can be attributed to variation within sampling positions. The rest of a small but highly significant amount of genetic variance corresponded to differences between countries (27.93%, *p* < .001) and among sampling sites within countries (11.99%, *p* < .001; Table 4).

**Table 4:** Results from the analyses of molecular variance (AMOVA) based on COI marker including the pooled China (18 sampling sites) and the USA samples

Source of variation	*df*	Sum of squares	Variance components	Variation (%)
Between countries	1	515.646	1.43395	27.93*
Among sampling sites within countries	17	369.464	0.61576	11.99***
Within sampling sites	740	2,282.728	3.08477	60.08***
Total	758	3,167.838	5.13447	

Abbreviations: *df*, degree of freedom.

* *p* < .05.

*** *p* < .001.

## 4 DISCUSSION

### 4.1 Suitable analysis method for *P. americana* identification

It is difficult to identify cockroaches by morphology due to several factors including phenotypic plasticity, developmental stochasticity, and sexual dimorphism. The molecular approach provides useful information that can be used for both identifying and defining the boundaries of species (Evangelista et al., 2013; Ruiz‐Lopez et al., 2012). OTUs obtained by applying the three methods (traditional tree‐based, ABGD, and GMYC) in our barcode data were compared. Traditional tree‐based delimitation approach with a 5.1% barcode gap was found to be more reliable and consistent in the identification of morphospecies when compared to the GMYC model and ABGD approach. GMYC analysis appeared to wrongly separate the *P. americana* morphospecies into two groupings (Figure 2a). A recent study (Camelier, Menezes, Costa‐Silva, & Oliveira, 2018) showed that GMYC typically generated a high number of OTUs than other methods. Errors in the ultrametric tree that underpins the analysis will lead to erroneous species identification (Zou et al., 2016).

Similar to the GMYC method, ABGD also oversplit *P. americana* into two candidate species with prior genetic distance thresholds between 0.77% and 1.29%. ABGD is a delimitation method based on genetic distances, intraspecific genetic variation and interspecific genetic divergence to congeners and would influence its delimitation accuracy (Pinto et al., 2015). *P. americana* samples in this study represent a mix of individuals from different locations within China and the United States. The maximum intraspecific COI sequence divergence within *P. americana* (5.1%) led to an overestimation of species diversity by the ABGD method (Hamilton, Hendrixson, Brewer, & Bond, 2014). When the *p* value ranged between 2.15% and 5.99%, ABGD placed *P. fuliginosa*, *P. australasiae*, and *P. sp* into a single candidate species and this was incongruent with the morphological evidence. Thus, the species delimited from ABGD analysis might be incorrect for the genetic distance threshold and can be produced by grouping closely related species into a single cluster, or even by separating relatively deep divergent populations into multiple clusters (Yu, Rao, Matsui, & Yang, 2017). ABGD is indeed influenced by the mix of shallow and deep genetic divergences.

We found that traditional tree‐based approach with a 5.1% barcode gap was a much better analytical method for *P. americana* identification. However, many researchers argued that DNA barcoding approaches are imperfect, and they cannot be used in species discovery and identification (Meyer & Paulay, 2005; Will & Rubinoff, 2004). No approach is the panacea to this problem. The shortcomings of DNA barcoding often mirror those approaches that rely strictly on morphological characteristics (Hamilton et al., 2014). The 5.1% intraspecific genetic variation within *P. americana* could accurately and effectively distinguish *P. americana* from other *Periplaneta* species in this study. This cutoff value could be applied to later research when diverse species are included, and as a supplement to traditional taxonomic techniques.

### 4.2 Low sequence variability and no genetic structure of *P. americana* populations

Population genetic structure and genetic diversity can provide important biological information for the study of invasive species (Wongsa, Duangphakdee, & Rattanawannee, 2017). However, there are limited studies on the population genetic structure of cockroaches (Cloarec, Rivault, & Cariou, 1999; Jaramillo‐Ramirez, Cárdenas‐Henao, González‐Obando, & Rosero‐Galindo, 2010; Vargo et al., 2014). Intraspecific phylogeny and haplotype networks all indicated the presence of very little genetic structure in *P. americana* (Figures 3 and 4). This result was in agreement with what was expected in the German cockroach (*Blattella germanica*; Vargo et al., 2014) and suggested that *P. americana* should be considered a global panmictic population (Troast, Suhling, Jinguji, Sahlén, & Ware, 2016). As one of the most widespread invasive insects, lack of population genetic structure of *P. americana* was likely due to a high reproductive rate and the human‐mediated range expansion of *P. americana* (Gonçalves et al., 2019; Vargo et al., 2014). AMOVA results for COI sequences in *P. americana* showed that most (>72%) of the genetic variation occurred within sampling sites and countries (Table 4), which indicated that gene flow was occurring on a global scale among *P. americana* groups from China and the United States. This high population admixture would lead to a wide spread of alleles and promote an increase in genetic diversity of invasive species (Gonçalves et al., 2019).

Unexpectedly, the relatively low genetic diversity within *P. americana* populations was observed in both mtDNA and nDNA markers. The analysis of COI and *wingless* fragments defined only 17 haplotypes from 741 individuals and 7 alleles from 113 individuals, respectively. Low population variation is common in other insects (Mazur et al., 2016; Žitko, Kovaćić, Desdevises, & Puizina, 2011). Several aspects can explain such a pattern of genetic variation: the small size of the founding populations (Žitko et al., 2011); the low genetic diversity in the original source populations (Vargo et al., 2014); and extensive insect control measures involving insecticides and source reduction (Prijović et al., 2014). Distinguishing between those possibilities will require the genetic characterization of one or more populations of *P. americana* native to Africa. However, the successful distribution of *P. americana* around the globe shows that invasive species with low genetic diversity can also be widely distributed and spread explosively (Wang, Li, & Wang, 2005).

### 4.3 Four main COI haplogroups of *P. americana*

Although no apparent phylogeographic assignment of mtDNA and nuclear lineages was observed in both BI trees, phylogenetic analyses based on *P. americana* COI haplotypes showed four divergent COI haplogroups (Figure 3). Mitochondrial DNA is inherited only through the maternal cytoplasm. Therefore, these four branches provide a record of the ancient maternal lineage of *P. americana*. In contrast to mtDNA, there is recombination in autosomal DNA. Recombination would distort the information on evolutionary history carried by the DNA sequence (Zhang & Hewitt, 2003), causing the discordance between the COI and *wingless* phylogenetic trees (Sota & Sasabe, 2006). The difference in rates of evolution between the mtDNA and nDNA may also be the reason of mito‐nuclear discordance. In insects, the evolution rates of mtDNA are estimated to be 2 ~ 9 times faster than nuclear protein‐coding regions (Lin & Danforth, 2004), and it may be insufficient to indicate phylogeographical patterns using nDNA with relatively low variation when compared to mtDNA (Hickerson & Cunningham, 2005). Thus, biogeographical analysis based on mtDNA was better suited in this study than nuclear DNA.

A number of successful applications of COI in phylogeographic studies have been reported (Inoue et al., 2013; Prijović et al., 2014; Qin et al., 2016). Haplotypes of the USA group were divided into three branches A, B, and D, while the Chinese group was divided into B, C, and D clades (Figure 3a). It appeared that groups of *P. americana* in China and the United States were each from three genetically divergent source groups, with a total of four clades. However, the rate of divergence for COI of Blattodea taxa was unknown, and a “universal” clock rate would cause error estimation in divergence time (Pfeiler, Bitler, Ramsey, Palacios‐Cardiel, & Markow, 2006). In future studies, a molecular clock should be applied to *P. americana* to estimate the ages of population genetic divergences in this species. Additionally, haplogroups B and D were shared between the USA and Chinese groups, as phylogenetic analysis did not exhibit obvious association between the haplotype phylogeny and geographic distribution (Figure 3). This may indicate a combination of historical admixture between groups (von Beeren et al., 2015), which is consistent with the century‐long global migration of this species (Schal, 2011). Frequent global trade and human‐mediated events likely presented advantageous conditions for the long‐distance dispersal of *P. americana*, which is considered to be ongoing.

## 5 CONCLUSION

This is the first study based on the COI gene to analyze the genetic diversity of *P. americana* groups in China. Taking into consideration the scale of individuals tested, it is also the largest phylogeographic study of this cockroach species. Although no clear pattern of genetic structure was detected within *P. americana*, four mitochondrial lineage units of *P. americana* showed clear genetic signatures of ancestor haplotypes. *P. americana* is a non‐native invasive species in China and is native to tropical Africa yet molecular evidence on the biogeographical origins of *P. americana* remains unresolved. Future researches should focus on collecting samples from African populations to better understand the origin of *P. americana* and its invasion history throughout the world. Our cockroach samples presented a mix of individuals from genetically distinct source groups. Despite these nongeographic groupings, our study showed that traditional tree‐based methods could accurately identify *P. americana* with a barcoding threshold of 5.1%. We believe that the mitochondrial COI gene can be effectively used in studying intra‐ and interspecific divergences of cockroaches.

## CONFLICT OF INTEREST

None declared.

## AUTHOR CONTRIBUTIONS

Zhang X conceived the project. Ma J and Liu J collected samples. Ma J, Liu J, and Shen Y performed the experiments and analyzed the data. Ma J, Fan Z, Zhang X, and Yue B wrote the manuscript with help from all of the authors.

## Data Availability

All COI and *wingless* sequences used in this study were deposited in the NCBI database under accession numbers: MF149138–MF149711, MH184206–MH184379, and MK658782–MK658829. The detailed information about the catalog of *Periplaneta* spp. specimens, COI genetic distance of *Periplaneta* haplotypes, and Fst and Nm values among different geographic groups of *Periplaneta americana* is available at the public Dryad Digital Repository: https://doi.org/10.5061/dryad.280gb5mm1.

## References

[ece35777-bib-0001] Akaike, H. (1974). A new look at the statistical model identification. IEEE Transactions on Automatic Control, 19(6), 716–723. 10.1109/TAC.1974.1100705

[ece35777-bib-0002] Beccaloni, G. W. (2014). Cockroach Species File Online. Version 5.0/5.0. World Wide Web electronic publication Retrieved from http://Cockroach.SpeciesFile.org. (cited 1 November 2018).

[ece35777-bib-0003] Bell, W. J. , Roth, L. M. , & Nalepa, C. A. (2007). Cockroaches: Ecology, behavior, and natural history. Baltimore, MD: Johns Hopkins University Press.

[ece35777-bib-0004] Camelier, P. , Menezes, N. A. , Costa‐Silva, G. J. , & Oliveira, C. (2018). Molecular and morphological data of the freshwater fish *Glandulocauda melanopleura* (Characiformes: Characidae) provide evidences of river captures and local differentiation in the Brazilian Atlantic Forest. PLoS One, 13(3), e0194247 10.1371/journal.pone.0194247 29579069 PMC5868800

[ece35777-bib-0005] Cameron, S. L. (2014). Insect mitochondrial genomics: Implications for evolution and phylogeny. Annual Review of Entomology, 59(1), 95–117. 10.1146/annurev-ento-011613-162007 24160435

[ece35777-bib-0006] Che, Y. , Gui, S. , Lo, N. , Ritchie, A. , & Wang, Z. (2017). Species delimitation and phylogenetic relationships in Ectobiid cockroaches (Dictyoptera, Blattodea) from China. PLoS One, 12(1), e0169006 10.1371/journal.pone.0169006 28046038 PMC5207705

[ece35777-bib-0007] Clement, M. , Posada, D. , & Crandall, K. A. (2000). TCS: A computer program to estimate gene genealogies. Molecular Ecology, 9(10), 1657–1659. 10.1046/j.1365-294x.2000.01020.x 11050560

[ece35777-bib-0008] Cloarec, A. , Rivault, C. , & Cariou, M. L. (1999). Genetic population structure of the German cockroach, *Blattella germanica*: Absence of geographical variation. Entomologia Experimentalis et Applicata, 92(3), 311–319. 10.1046/j.1570-7458.1999.00552.x

[ece35777-bib-0009] Evangelista, D. A. , Bourne, G. , & Ware, J. L. (2014). Species richness estimates of Blattodea s.s. (Insecta: Dictyoptera) from northern Guyana vary depending upon methods of species delimitation. Systematic Entomology, 39(1), 150–158. 10.1111/syen.12043

[ece35777-bib-0010] Evangelista, D. , Buss, L. , & Ware, J. L. (2013). Using DNA barcodes to confirm the presence of a new invasive cockroach pest in New York City. Journal of Economic Entomology, 106(6), 2275–2279. 10.1603/EC13402 24498724

[ece35777-bib-0011] Excoffier, L. , & Lischer, H. E. L. (2010). Arlequin suite ver 3.5: A new series of programs to perform population genetics analyses under Linux and Windows. Molecular Ecology Resources, 10(3), 564–567. 10.1111/j.1755-0998.2010.02847.x 21565059

[ece35777-bib-0012] Ferronato, P. , Woch, A. L. , Soares, P. L. , Bernardi, D. , Botton, M. , Andreazza, F. , … Corrêa, A. S. (2019). A phylogeographic approach to the *Drosophila suzukii* (Diptera: Drosophilidae) Invasion in Brazil. Journal of Economic Entomology, 112(1), 425–433. 10.1093/jee/toy321 30383249

[ece35777-bib-0013] Folmer, O. , Black, M. , Hoeh, W. , Lutz, R. , & Vrijenhoek, R. (1994). DNA primers for amplification of mitochondrial cytochrome c oxidase subunit I from diverse metazoan invertebrates. Molecular Marine Biology and Biotechnology, 3(5), 294–299. 7881515

[ece35777-bib-0014] Fujisawa, T. , & Barraclough, T. G. (2013). delimiting species using single‐locus data and the generalized mixed yule coalescent approach: A revised method and evaluation on simulated data sets. Systematic Biology, 62(5), 707–724. 10.1093/sysbio/syt033 23681854 PMC3739884

[ece35777-bib-0015] Gonçalves, R. M. , Mastrangelo, T. , Rodrigues, J. C. V. , Paulo, D. F. , Omoto, C. , Corrêa, A. S. , & de Azeredo‐Espin, A. M. L. (2019). Invasion origin, rapid population expansion, and the lack of genetic structure of cotton bollworm (*Helicoverpa armigera*) in the Americas. Ecology and Evolution, 9(13), 7378–7401. 10.1002/ece3.5123 31346410 PMC6635935

[ece35777-bib-0016] Hamilton, C. A. , Hendrixson, B. E. , Brewer, M. S. , & Bond, J. E. (2014). An evaluation of sampling effects on multiple DNA barcoding methods leads to an integrative approach for delimiting species: A case study of the North American tarantula genus *Aphonopelma* (Araneae, Mygalomorphae, Theraphosidae). Molecular Phylogenetics and Evolution, 71, 79–93. 10.1016/J.YMPEV.2013.11.007 24280211

[ece35777-bib-0017] Hickerson, M. J. , & Cunningham, C. W. (2005). Contrasting quaternary histories in an ecologically divergent sister pair of low‐dispersing intertidal fish (Xiphister) revealed by multilocus DNA analysis. Evolution, 59, 344–360. 10.1111/j.0014-3820.2005.tb00994.x 15807420

[ece35777-bib-0018] Inoue, M. N. , Sunamura, E. , Suhr, E. L. , Ito, F. , Tatsuki, S. , & Goka, K. (2013). Recent range expansion of the Argentine ant in Japan. Diversity and Distributions, 19(1), 29–37. 10.1111/j.1472-4642.2012.00934.x

[ece35777-bib-0019] Jaramillo‐Ramirez, G. I. , Cárdenas‐Henao, H. , González‐Obando, R. , & Rosero‐Galindo, C. Y. (2010). Genetic variability of Five *Periplaneta americana* L. (Dyctioptera: Blattidae) populations in southwestern Colombia using the AFLP molecular marker technique. Neotropical Entomology, 39(3), 371–378. 10.1590/S1519-566X2010000300010 20676510

[ece35777-bib-0020] Johnson, A. J. , Morton, P. K. , Schemerhorn, B. J. , & Shukle, R. H. (2011). Use of a nuclear marker to assess population structure in Hessian Fly (Diptera: Cecidomyiidae). Annals of the Entomological Society of America, 104(4), 666–674. 10.1603/AN10154

[ece35777-bib-0021] Librado, P. , & Rozas, J. (2009). DnaSP v5: A software for comprehensive analysis of DNA polymorphism data. Bioinformatics, 25(11), 1451–1452. 10.1093/bioinformatics/btp187 19346325

[ece35777-bib-0022] Lin, C.‐P. , & Danforth, B. N. (2004). How do insect nuclear and mitochondrial gene substitution patterns differ? Insights from Bayesian analyses of combined datasets. Molecular Phylogenetics and Evolution, 30(3), 686–702. 10.1016/S1055-7903(03)00241-0 15012948

[ece35777-bib-0023] Liu, X. W. , Zhu, W. B. , Da, L. , & Wang, H. Q. (2017). Cockroach in southeast China. Zhengzhou, China: Henan science and technology press.

[ece35777-bib-0024] Mazur, M. A. , Holecová, M. , Lachowska‐Cierlik, D. , Lis, A. , Kubisz, D. , & Kajtoch, Ł. (2016). Selective sweep of *Wolbachia* and parthenogenetic host genomes – The example of the weevil *Eusomus ovulum* . Insect Molecular Biology, 25(6), 701–711. 10.1111/imb.12255 27438898

[ece35777-bib-0025] Meyer, C. P. , & Paulay, G. (2005). DNA Barcoding: Error rates based on comprehensive sampling. PLoS Biology, 3(12), e422 10.1371/journal.pbio.0030422 16336051 PMC1287506

[ece35777-bib-0026] Ni, L. , Li, Q. , Kong, L. , Huang, S. , & Li, L. (2012). DNA barcoding and phylogeny in the family Mactridae (Bivalvia: Heterodonta): Evidence for cryptic species. Biochemical Systematics and Ecology, 44, 164–172. 10.1016/j.bse.2012.05.008

[ece35777-bib-0027] Pfeiler, E. , Bitler, B. G. , Ramsey, J. M. , Palacios‐Cardiel, C. , & Markow, T. A. (2006). Genetic variation, population structure, and phylogenetic relationships of *Triatoma rubida* and *T. recurva* (Hemiptera: Reduviidae: Triatominae) from the Sonoran Desert, insect vectors of the Chagas' disease parasite *Trypanosoma cruzi* . Molecular Phylogenetics and Evolution, 41(1), 209–221. 10.1016/j.ympev.2006.07.001 16934496

[ece35777-bib-0028] Pinto, I. D. S. , Chagas, B. D. D. , Rodrigues, A. A. F. , Ferreira, A. L. , Rezende, H. R. , Bruno, R. V. , … Peixoto, A. A. (2015). DNA barcoding of Neotropical sand flies (Diptera, Psychodidae, Phlebotominae): Species identification and discovery within Brazil. PLoS One, 10(10), e0140636 10.1371/journal.pone.0140636 26506007 PMC4624639

[ece35777-bib-0029] Posada, D. , & Crandall, K. A. (1998). MODELTEST: Testing the model of DNA substitution. Bioinformatics, 14(9), 817–818. 10.1093/bioinformatics/14.9.817 9918953

[ece35777-bib-0030] Prijović, M. , Škaljac, M. , Drobnjaković, T. , Žanić, K. , Perić, P. , Marčić, D. , & Puizina, J. (2014). Genetic variation of the greenhouse whitefly, *Trialeurodes vaporariorum* (Hemiptera: Aleyrodidae), among populations from Serbia and neighbouring countries, as inferred from COI sequence variability. Bulletin of Entomological Research, 104(3), 357–366. 10.1017/S0007485314000169 24661625

[ece35777-bib-0031] Puillandre, N. , Lambert, A. , Brouillet, S. , & Achaz, G. (2012). ABGD, Automatic Barcode Gap Discovery for primary species delimitation. Molecular Ecology, 21(8), 1864–1877. 10.1111/j.1365-294X.2011.05239.x 21883587

[ece35777-bib-0032] Qin, Y.‐J. , Buahom, N. , Krosch, M. N. , Du, Y. U. , Wu, Y. I. , Malacrida, A. R. , … Li, Z.‐H. (2016). Genetic diversity and population structure in *Bactrocera correcta* (Diptera: Tephritidae) inferred from mtDNA cox1 and microsatellite markers. Scientific Reports, 6(1), 38476 10.1038/srep38476 27929126 PMC5144084

[ece35777-bib-0033] Rambaut, A. (2007). FigTree, a graphical viewer of phylogenetic trees. Retrieved from http://treebioed.ac.uk/software/figtree (cited 1 November 2018).

[ece35777-bib-0034] Robinson, W. H. (2005). Urban insects and Arachnids – A handbook of urban entomology. Cambridge, UK: Cambridge University Press.

[ece35777-bib-0035] Roman, J. (2006). Diluting the founder effect: Cryptic invasions expand a marine invader's range. Proceedings of the Royal Society B: Biological Sciences, 273(1600), 2453–2459. 10.1098/rspb.2006.3597 PMC1634897 16959635

[ece35777-bib-0036] Ronquist, F. , & Huelsenbeck, J. P. (2003). MrBayes 3: Bayesian phylogenetic inference under mixed models. Bioinformatics, 19(12), 1572–1574. 10.1093/bioinformatics/btg180 12912839

[ece35777-bib-0037] Roth, L. M. , & Willis, E. R. (1960). The biotic associations of cockroaches. Smithsonian Miscellaneous Collections, 141, 1–470. 10.1002/jps.2600500438

[ece35777-bib-0038] Ruiz‐Lopez, F. , Wilkerson, R. C. , Conn, J. E. , McKeon, S. N. , Levin, D. M. , Quiñones, M. L. , … Linton, Y.‐M. (2012). DNA barcoding reveals both known and novel taxa in the Albitarsis Group (Anopheles: Nyssorhynchus) of Neotropical malaria vectors. Parasites & Vectors, 5(1), 44 10.1186/1756-3305-5-44 22353437 PMC3350407

[ece35777-bib-0039] Schal, C. (2011). Cockroaches In Hedges S., & Moreland D. (Eds.), Mallis handbook of pest control (10th ed., pp. 150–291). Cleveland, OH: GIE Media.

[ece35777-bib-0040] Simon, C. , Buckley, T. R. , Frati, F. , Stewart, J. B. , & Beckenbach, A. T. (2006). Incorporating molecular evolution into phylogenetic analysis, and a new compilation of conserved polymerase chain reaction primers for animal mitochondrial DNA. Annual Review of Ecology, Evolution, and Systematics, 37(1), 545–579. 10.1146/annurev.ecolsys.37.091305.110018

[ece35777-bib-0041] Song, H. , Moulton, M. J. , & Whiting, M. F. (2014). Rampant nuclear insertion of mtDNA across diverse lineages within Orthoptera (Insecta). PLoS One, 9(10), e110508 10.1371/journal.pone.0110508 25333882 PMC4204883

[ece35777-bib-0042] Sota, T. , & Sasabe, M. (2006). Utility of nuclear allele networks for the analysis of closely related species in the genus *Carabus* subgenus *Ohomopterus* . Systematic Biology, 55(2), 329–344. 10.1080/10635150500541607 16611603

[ece35777-bib-0043] Suchard, M. A. , Lemey, P. , Baele, G. , Ayres, D. L. , Drummond, A. J. , & Rambaut, A. (2018). Bayesian phylogenetic and phylodynamic data integration using BEAST 1.10. Virus Evolution, 4(1), vey016 10.1093/ve/vey016 29942656 PMC6007674

[ece35777-bib-0044] Talavera, S. , Muñoz‐Muñoz, F. , Verdún, M. , & Pagès, N. (2017). Morphology and DNA barcoding reveal three species in one: Description of *Culicoides cryptipulicaris* sp. nov. and *Culicoides quasipulicaris* sp. nov. in the subgenus *Culicoides* . Medical and Veterinary Entomology, 31(2), 178–191. 10.1111/mve.12228 28370147

[ece35777-bib-0045] Tamura, K. , Peterson, D. , Peterson, N. , Stecher, G. , Nei, M. , & Kumar, S. (2011). MEGA5: Molecular evolutionary genetics analysis using maximum likelihood, evolutionary distance, and maximum parsimony methods. Molecular Biology and Evolution, 28(10), 2731–2739. 10.1093/molbev/msr121 21546353 PMC3203626

[ece35777-bib-0046] Troast, D. , Suhling, F. , Jinguji, H. , Sahlén, G. , & Ware, J. (2016). A global population genetic study of *Pantala flavescens* . PLoS One, 11(3), e0148949 10.1371/journal.pone.0148949 26934181 PMC4775058

[ece35777-bib-0047] Vargo, E. L. , Crissman, J. R. , Booth, W. , Santangelo, R. G. , Mukha, D. V. , & Schal, C. (2014). Hierarchical genetic analysis of German Cockroach (*Blattella germanica*) populations from within buildings to across continents. PLoS One, 9(7), e102321 10.1371/journal.pone.0102321 25020136 PMC4096728

[ece35777-bib-0048] Versteirt, V. , Nagy, Z. T. , Roelants, P. , Denis, L. , Breman, F. C. , Damiens, D. , … Van Bortel, W. (2015). Identification of Belgian mosquito species (Diptera: Culicidae) by DNA barcoding. Molecular Ecology Resources, 15(2), 449–457. 10.1111/1755-0998.12318 25143182

[ece35777-bib-0049] von Beeren, C. , Stoeckle, M. Y. , Xia, J. , Burke, G. , & Kronauer, D. J. C. (2015). Interbreeding among deeply divergent mitochondrial lineages in the American cockroach (*Periplaneta americana*). Scientific Reports, 5(1), 8297 10.1038/srep08297 25656854 PMC4650827

[ece35777-bib-0050] Wang, B. , Li, W. , & Wang, J. (2005). Genetic diversity of *Alternanthera philoxeroides* in China. Aquatic Botany, 81(3), 277–283. 10.1016/J.AQUABOT.2005.01.004

[ece35777-bib-0051] Wang, C. , Li, S. , Fu, C. , Gong, X. , Huang, L. , Song, X. , & Zhao, Y. (2009). Molecular genetic structure and evolution in native and colonized populations of the Chinese mitten crab, *Eriocheir sinensis* . Biological Invasions, 11(2), 389–399. 10.1007/s10530-008-9256-8

[ece35777-bib-0052] Will, K. W. , & Rubinoff, D. (2004). Myth of the molecule: DNA barcodes for species cannot replace morphology for identification and classification. Cladistics, 20(1), 47–55. 10.1111/j.1096-0031.2003.00008.x 34892971

[ece35777-bib-0053] Wongsa, K. , Duangphakdee, O. , & Rattanawannee, A. (2017). Genetic structure of the *Aphis craccivora* (Hemiptera: Aphididae) from Thailand inferred from mitochondrial COI gene sequence. Journal of Insect Science, 17(4), 84 10.1093/jisesa/iex058 PMC5510963 28973491

[ece35777-bib-0054] Yu, G. , Rao, D. , Matsui, M. , & Yang, J. (2017). Coalescent‐based delimitation outperforms distance‐based methods for delineating less divergent species: The case of *Kurixalus odontotarsus* species group. Scientific Reports, 7(1), 16124 10.1038/s41598-017-16309-1 29170403 PMC5700917

[ece35777-bib-0055] Zhang, D. X. , & Hewitt, G. M. (1996). Nuclear integrations: Challenges for mitochondrial DNA markers. Trends in Ecology & Evolution, 11(6), 247–251. 10.1016/0169-5347(96)10031-8 21237827

[ece35777-bib-0056] Zhang, D. X. , & Hewitt, G. M. (2003). Nuclear DNA analyses in genetic studies of populations: Practice, problems and prospects. Molecular Ecology, 12(3), 563–584. 10.1046/j.1365-294X.2003.01773.x 12675814

[ece35777-bib-0057] Žitko, T. , Kovaćić, A. , Desdevises, Y. , & Puizina, J. (2011). Genetic variation in east‐adriatic populations of the Asian tiger mosquito, *Aedes albopictus* (Diptera: Culicidae), inferred from NADH5 and COI sequence variability. European Journal of Entomology, 108(4), 501–508. 10.14411/eje.2011.065

[ece35777-bib-0058] Zou, S. , Fei, C. , Wang, C. , Gao, Z. , Bao, Y. , He, M. , & Wang, C. (2016). How DNA barcoding can be more effective in microalgae identification: A case of cryptic diversity revelation in Scenedesmus (Chlorophyceae). Scientific Reports, 6(1), 36822 10.1038/srep36822 27827440 PMC5101840

