# Supplementary material for: Population genetic structure and intraspecific genetic distance of Periplaneta americana (Blattodea: Blattidae) based on mitochondrial and nuclear DNA markers
Source: Ecol Evol. 2019 Nov 4;9(22):12928–39. doi: 10.1002/ece3.5777 (PMC6876684; doi:10.1002/ece3.5777)
Supplement: Supplementary file 1 [file ECE3-9-12928-s001.docx]

Table S1 Sample information of *Periplaneta* cockroaches used in this study. Abbreviations: *Periplaneta americana* (PA), *Periplaneta fuliginosa* (FU), *Periplaneta australasiae* (AU), *Periplaneta brunnea* (BR)*, Periplaneta* sp. (SP), *Periplaneta japonica* (JA).

| Species | Collection site | No. of location | Locality ID | Haplotype | No. of specimens |
| --- | --- | --- | --- | --- | --- |
| *Periplaneta americana* | Xichang, Sichuan, China (farm cultured) (27°55’, 102°13’) | 1 | PAXC | PAH1(58); PAH2(52); PAH4(9); PAH6(1) | 120 |
|  | Rongxian, Zigong, Sichuan, China (farm cultured) (29°26’, 104°24’) | 2 | PAZG | PAH1(19); PAH2(9); PAH5(1); PAH7(1) | 30 |
|  | Xuancheng, Anhui, China (farm cultured) (30°56’, 118°45’) | 3 | PAAH | PAH1(24); PAH2(5); PAH3(1) | 30 |
|  | Huaian, Jiangsu, China (farm cultured) (33°36’, 119°00’) | 4 | PAHA | PAH1(14); PAH2(12); PAH4(4) | 30 |
|  | Yangjiang, Guangdong, China (farm cultured) (21°51’, 111°59’) | 5 | PAYJ | PAH1(10); PAH2(17); PAH4(2); PAH5(1) | 30 |
|  | Wenzhou, Zhejiang, China (farm cultured) (27°59’, 120°41’) | 6 | PAWZ | PAH1(30) | 30 |
|  | Chongqiong, China (farm cultured) (29°33’, 106°33’) | 7 | PACQ | PAH1(30) | 30 |
|  | Dali, Yunnan, China (farm cultured) (25°36’, 100°15’) | 8 | PADL | PAH1(21); PAH4(3); PAH2(6) | 30 |
|  | Feicheng, Shandong, China (farm cultured) (36°10’, 116°46’) | 9 | PATA | PAH1(31) | 31 |
|  | Qiaocheng District, Bozhou, Anhui, China (farm cultured) (33°52’, 115°46’) | 25 | PAQC | PAH1(30) | 30 |
|  | Guangzhou, Guangdong, China (23°07’, 113°15’) | 10 | PAGZ | PAH1(8) | 8 |
|  | Chengdu, Sichuan, China (30°34’, 104°03’) | 11 | PACD | PAH1(33) | 33 |
|  | Hezhou, Guangxi, China (24°23’, 111°34’) | 31 | PAHZ | PAH1(32) | 32 |
|  | Chuxiong,Yunnan, China (25°02’, 101°31’) | 24 | PACX | PAH1(34) | 34 |
|  | Dongguan, Guangdong, China (22°56’, 113°40’) | 22 | PAHM | PAH1(29); PAH8(1) | 30 |
|  | Fujian Normal University, Fuzhou, Fujian, China (26°1’, 119°12’) | 27 | PAFZ | PAH1(26); PAH9(4) | 30 |
|  | Yantian District, Shenzhen, Guangdong, China (22°33’, 114°14’) | 29 | PASZ | PAH1(5) | 5 |
|  | Zhongshang, Guangdong, China (22°34’, 113°32’)* | N/A | PACN | PAH10(1) | 1 |
|  | USA* | N/A | PAUS | PAH1(44); PAH11(29); PAH12(48); PAH13(44); PAH14(4); PAH15(16); PAH16(1); PAH17(9) | 195 |
| *Periplaneta fuliginosa* | Chengdu, Sichuan, China (30°34’, 104°03’) | 11 | FUCD | FUH1(1); FUH2(1); FUH3(58); FUH4(4) | 64 |
|  | Yibin, Sichuan, China (28°45’, 104°38’) | 12 | FUYB | FUH3(3); FUH4(3) | 6 |
|  | Leshan, Sichuan, China (29°33’, 103°45’) | 13 | FULS | FUH3(3); FUH4(3); FUH7(2); FUH8(1) | 9 |
|  | Yingshan, Nanchong, Sichuan, China (31°04’, 106°34’) | 14 | FUYS | FUH3(1) | 1 |
|  | Liziping, Shimian, Ya'an, Sichuan, China (29°13’, 102°21’) | 15 | FUSM | FUH10(1) | 1 |
|  | Langzhong, Nanchong, Sichuan, China (31°33’, 106°00’) | 16 | FULZ | FUH3(3) | 3 |
|  | Feishui, Mianyang, Sichuan, China (31°32’, 104°34’) | 17 | FUMY | FUH3(29); FUH9(1) | 30 |
|  | Kunming, Yunnan, China (24°53’, 102°50’) | 18 | FUKM | FUH3(31); FUH6(2) | 33 |
|  | Luliang, Qujing, Yunnan, China (25°01’, 103°39’) | 19 | FULN | FUH3(19) | 19 |
|  | Shuangfeng, Loudi, Hunan, China (27°27’, 112°10’) | 20 | FUHN | FUH3(6); FUH5(2) | 8 |
|  | Guangnan, Wenshan, Yunnan, China (24°2’, 105°3’) | 26 | FUGN | FUH11(1) | 1 |
|  | Weixin, Zhaotong, Yunnan, China (27°50’, 105°2’) | 28 | FUZT | FUH3(23); FUH8(7) | 30 |
|  | Xinshao, Shaoyang, Hunan, China (27°31’, 111°15’) | 30 | FUSY | FUH3(30) | 30 |
| *Periplaneta australasiae* | Bobai, Yulin, Guangxi, China (22°16’, 109°58’) | 21 | AUYL | AUH1(3); AUH2(1) | 4 |
|  | Houjie, Dongguan, Guangdong, China (22°56’, 113°40’) | 22 | AUDG | AUH1(5); AUH3(1) | 6 |
|  | Huaning, Yuxi, Yunnan, China (24°11’, 102°55’) | 23 | AUYX | AUH1(1); AUH2(1); AUH4(28) | 30 |
|  | Chuxiong, Yunnan, China (25°02’, 101°31’) | 24 | AUCX | AUH1(5) | 5 |
|  | Fujian Normal University, Fuzhou, Fujian, China (26°1’, 119°12’) | 27 | AUFJ | AUH5(1) | 1 |
|  | Yantian District, Shenzhen, Guangdong, China (22°33’, 114°14’) | 29 | AUSZ | AUH1(3); AUH3(3) | 6 |
| *Periplaneta brunnea* | Guangzhou, Guangdong, China (23°07’, 113°15’) | 10 | BRGZ | BRH1(2); BRH2(1) | 3 |
| *Periplaneta* sp. | USA* | N/A | SPUS | SPH1(1) | 1 |
| *Periplaneta japonica* | Korea* | N/A | JAKO | JAH1(3) | 3 |

*Source from GenBank, N/A = not applicable.
