# Supplementary material for: Population genetic structure and intraspecific genetic distance of Periplaneta americana (Blattodea: Blattidae) based on mitochondrial and nuclear DNA markers
Source: Ecol Evol. 2019 Nov 4;9(22):12928–39. doi: 10.1002/ece3.5777 (PMC6876684; doi:10.1002/ece3.5777)
Supplement: Supplementary file 2 [file ECE3-9-12928-s002.docx]

Table S2 Summary of molecular diversity indices and group expansion test statistics of mitochondrial COI and *wingless* genes of *Periplaneta americana.* Detailed information on sampling localities are as indicated in Table S1.

| Marker | Collection country | Locality ID | Number of individuals | Haplotype/Allele Number | Haplotype  Diversity (Hd) | Nucleotide Diversity (Pi) |
| --- | --- | --- | --- | --- | --- | --- |
| COI | China | PAXC | 120 | PAH1(58); PAH2(52); PAH4(9); PAH6(1) | 0.578 | 0.01061 |
|  |  | PAZG | 30 | PAH1(19); PAH2(9); PAH5(1); PAH7(1) | 0.524 | 0.00859 |
|  |  | PAAH | 30 | PAH1(24); PAH2(5); PAH3(1) | 0.343 | 0.00534 |
|  |  | PAHA | 30 | PAH1(14); PAH2(12); PAH4(4) | 0.625 | 0.01179 |
|  |  | PAYJ | 30 | PAH1(10); PAH2(17); PAH4(2); PAH5(1) | 0.582 | 0.01014 |
|  |  | PAQC | 30 | PAH1(30) | 0 | 0 |
|  |  | PADL | 30 | PAH1(21); PAH4(3); PAH2(6) | 0.476 | 0.00912 |
|  |  | PATA | 31 | PAH1(31) | 0 | 0 |
|  |  | PAWZ | 30 | PAH1(30) | 0 | 0 |
|  |  | PACQ | 30 | PAH1(30) | 0 | 0 |
|  |  | PACD | 33 | PAH1(33) | 0 | 0 |
|  |  | PAHZ | 32 | PAH1(32) | 0 | 0 |
|  |  | PACX | 34 | PAH1(34) | 0 | 0 |
|  |  | PAHM | 30 | PAH1(29); PAH8(1) | 0.067 | 0.00030 |
|  |  | PAFZ | 30 | PAH1(26); PAH9(4) | 0.239 | 0.00036 |
|  |  | PASZ | 5 | PAH1(5) | 0 | 0 |
|  |  | PAGZ | 8 | PAH1(8) | 0 | 0 |
|  |  | PACN | 1 | PAH10(1) | 0 | 0 |
|  | **Total in China** | - | 564 | PAH1(434); PAH2(101); PAH3(1); PAH4(18); PAH5(2); PAH6(1); PAH7(1); PAH8(1); PAH9(4); PAH10(1) | 0.375 | 0.00659 |
|  | USA | PAUS | 195 | PAH1(44); PAH11(29); PAH12(48); PAH13(44); PAH14(4); PAH15(16); PAH16(1); PAH17(9) | 0.810 | 0.02243 |
| *wingless* | China | - | 48 | Allele1/1(11); Allele1/5(3); Allele2/2(19); Allele2/4(2); Allele2/5(10); Allele2/7(1); Allele5/5(2) | 0.625 | 0.00544 |
|  | USA | - | 65^a^ | Allele1/3(1); Allele1/4(5); Allele1/7(2); Allele2/3(1); Allele2/4(2); Allele2/5(1); Allele2/7(4); Allele3/3(2); Allele3/4(3); Allele3/5(1); Allele3/7(6); Allele4/4(13); Allele4/5(3); Allele4/6(1);  Allele4/7(10); Allele5/7(1); Allele6/7(1); Allele7/7(8) | 0.738 | 0.01000 |

a. Three individuals (KM591680.1, KM591631.1, KM591621.1) were omitted for this analysis for their inferred alleles with low probability (P<0.8).
