# Supplementary figures and images for: Population genetic structure and intraspecific genetic distance of Periplaneta americana (Blattodea: Blattidae) based on mitochondrial and nuclear DNA markers
Source: Ecol Evol. 2019 Nov 4;9(22):12928–39. doi: 10.1002/ece3.5777 (PMC6876684; doi:10.1002/ece3.5777)

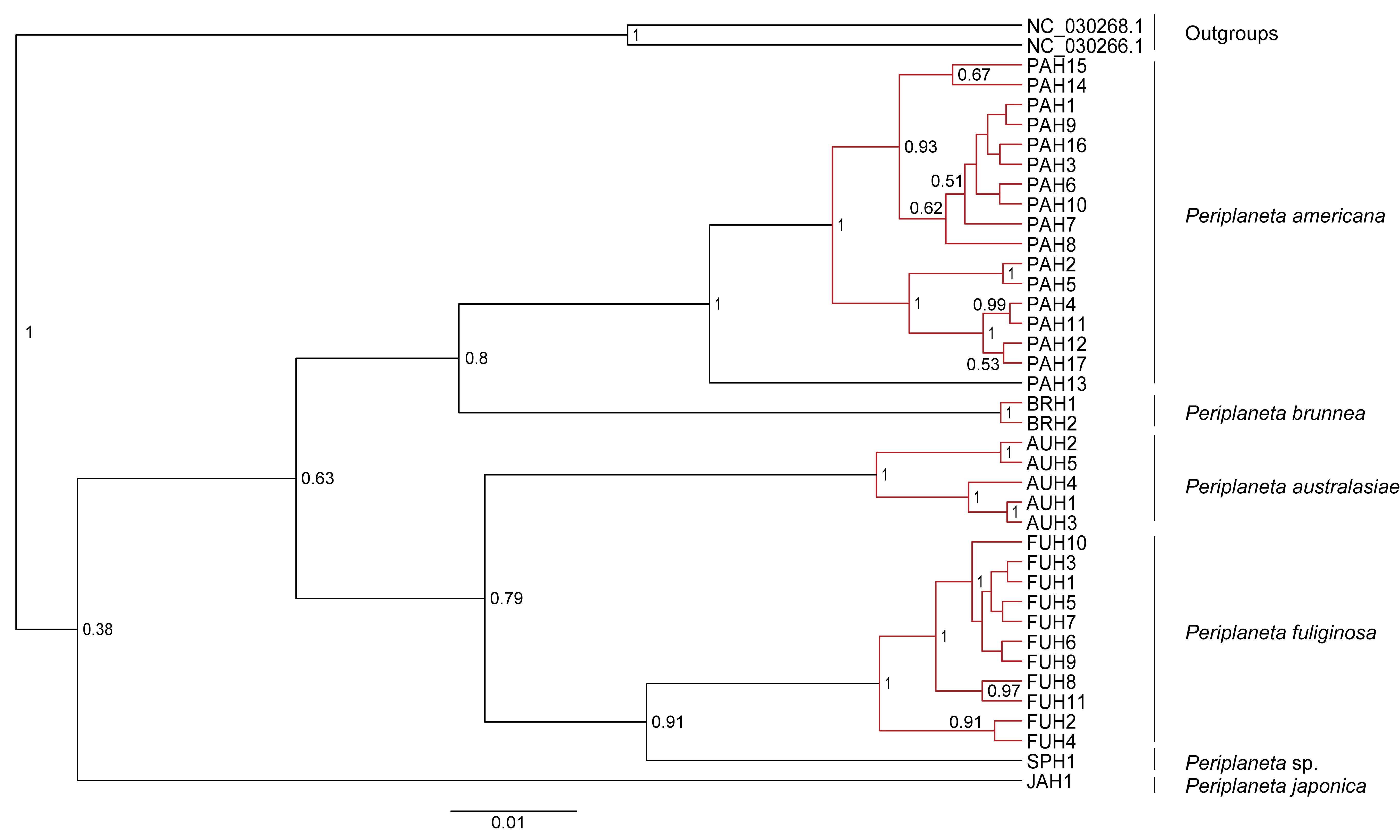

Supplement: Supplementary file 3 [file ECE3-9-12928-s003.tif]
