# Supplementary material for: Population genetic structure and intraspecific genetic distance of Periplaneta americana (Blattodea: Blattidae) based on mitochondrial and nuclear DNA markers
Source: Ecol Evol. 2019 Nov 4;9(22):12928–39. doi: 10.1002/ece3.5777 (PMC6876684; doi:10.1002/ece3.5777)
Supplement: Supplementary file 4 [file ECE3-9-12928-s004.docx]

APPENDIX S3 Ultrametric tree obtained with beast using the COI alignment. Generalized mixed Yule-Coalescent model genetic clusters recognized as species are highlighted in red. The numbers in the nodes represent the posterior values, and the values exceeding 50% are shown above the nodes.
